# Heat tolerance and thermal preference of the copepod *Tigriopus californicus* are insensitive to ecologically relevant dissolved oxygen levels

**DOI:** 10.1038/s41598-020-75635-z

**Published:** 2020-11-03

**Authors:** Khuong V. Dinh, Arani Y. Cuevas-Sanchez, Katherine S. Buhl, Elizabeth A. Moeser, W. Wesley Dowd

**Affiliations:** 1 School of Biological Sciences, Washington State University, P.O. Box 644236, Pullman, WA 99164-4236 USA; 2 Department of Environmental Studies, University of Southern California, Los Angeles, CA, USA

**Keywords:** Behavioural ecology, Ecophysiology

## Abstract

Shifting climate patterns may impose novel combinations of abiotic conditions on animals, yet understanding of the present-day interactive effects of multiple stressors remains under-developed. We tested the oxygen and capacity limited thermal tolerance (OCLTT) hypothesis and quantified environmental preference of the copepod *Tigriopus californicus*, which inhabits rocky-shore splashpools where diel fluctuations of temperature and dissolved oxygen (DO) are substantial. Egg-mass bearing females were exposed to a 5 h heat ramp to peak temperatures of 34.1–38.0 °C crossed with each of four oxygen levels: 22, 30, 100 and 250% saturation (4.7–5.3, 5.3–6.4, 21.2–21.3, and 50.7–53.3 kPa). Survival decreased at higher temperatures but was independent of DO. The behavioral preference of females was quantified in seven combinations of gradients of both temperature (11–37 °C) and oxygen saturation (17–206% or 3.6–43.6 kPa). Females avoided high temperatures regardless of DO levels. This pattern was more pronounced when low DO coincided with high temperature. In uniform temperature treatments, the distribution shifted toward high DO levels, especially in uniform high temperature, confirming that *Tigriopus* can sense environmental *p*O_2_. These results question the ecological relevance of OCLTT for *Tigriopus* and raise the possibility of microhabitat selection being used within splashpool environments to avoid physiologically stressful combinations of conditions.

## Introduction

Marine organisms are facing increasing prevalence of multiple stressors, particularly extreme temperatures and heatwaves^1–5^ and low dissolved oxygen^6–9^. Furthermore, stressors can interact and modulate each other’s effects^10–12^. For example, extreme temperatures decrease the oxygen solubility while simultaneously increasing the organism’s oxygen demand, hence intensifying hypoxic stress on marine species^7,13^. To locally survive, organisms may have to adjust their physiology^14–16^ and/or behaviors^14,17,18^ to cope with or avoid stressful conditions. However, we know relatively little about the prevalence and magnitude of the present-day interactions among stressors and their impacts on marine species^10–12^.

A prevailing theory suggests that thermal tolerance, particularly of aquatic water-breathers, is dependent upon oxygen availability. Specifically, this theory posits that thermal tolerance is determined by the capacity for oxygen supply in relation to oxygen demand; these ideas are encapsulated in the Oxygen and Capacity Limited Thermal Tolerance hypothesis (OCLTT)^19–21^. There are physiological studies supporting OCLTT, particularly during acute thermal stress events. For example, the critical thermal maxima (CT_max_) of fish increases under hyperoxia^22^. Yet, there is increasing evidence questioning the generality and ecological relevance of the OCLTT hypothesis^23–26^. For example, Lehmann et al.^27^ found that CT_max_ of the pupae of the butterfly *Pieris napi* was 43.1 ± 0.5 °C, and it did not change across the range of ambient *p*O_2_ from 10 to 30 kPa. Similarly, the heat tolerance of grasshopper larvae (*Schistocerca americana*) was not decreased under hypoxic conditions^25^. These counter-examples often come from terrestrial systems, but recent work also questions the predictions of OCLTT in water-breathers such as fish^4^.

By similar logic, it is also possible that oxygen levels influence organismal temperature preferences. Mobile species can employ behavioral adjustments to avoid stressful conditions by selecting specific microhabitats in spatially heterogeneous environments^14,18^. Indeed, spatial heterogeneity is a widespread feature of natural habitats^28,29^, even in small splashpools^29,30^. Behavioral adjustments can provide an alternative mechanism to rescue species from stressful conditions^14,17,18^. Many species can adjust their position to seek preferred thermal and oxygen zones^31,32^. For example, the American lobster (*Homarus americanus*) responded to changes in water temperature by leaving their shelter and seeking temperatures within ~ 1.2 °C of the acclimation temperature^31^. Similarly, *Cyclops vicinus* copepods showed their highest distribution (presence) within their optimal thermal conditions^33^. Calanoid copepod species prefer oxygen-rich water while cyclopoid *Oihona similis* and *Oncaea* sp. often aggregate in high abundance in the midwater hypoxic zone^32^. Fewer studies have examined preference behaviors in a multi-stressor context, and those showed that fish have a lower temperature preference in hypoxic conditions than in normoxia^34,35^. Overlooking the potentials of behavioral responses to multiple stressors such as extreme temperatures and low dissolved oxygen (DO) levels may fail to accurately quantify the real risk of environmental changes in natural populations^17,18^.

Harpacticoid copepods in the genus *Tigriopus* are excellent species to study physiological and behavioral responses to single and covaring stressors. These species are commonly found in splashpools^36–38^, where the diel fluctuations of temperatures and DO are substantial^30^. High temperatures (> 30 °C) tend to coincide with high DO levels (> 200% saturation) during the day due to the elevated solar radiation and photosynthesis; low temperatures and low DO conditions are observed during the night^30^. *Tigriopus* spp. show a high tolerance to temperatures^39–41^ and low DO^42–44^ independently. However, the majority of these studies have investigated the impact of one stressor at a time, thereby leaving unexplored the potential for interactive effects that might impact organisms experiencing multiple stressors simultaneously in nature^15^. The behavioral responses of *Tigriopus* to temperature, dissolved oxygen, and their combination remain to be tested.

In this study, we combined physiological and behavioral tests to comprehensively assess whether (1) ecologically relevant high (low) oxygen levels may increase (decrease) heat tolerance of *Tigriopus californicus*, as predicted by the OCLTT hypothesis, and (2) copepods modulate their thermal preference depending on oxygen levels.

## Results

### Thermal tolerance

**Figure 1:**
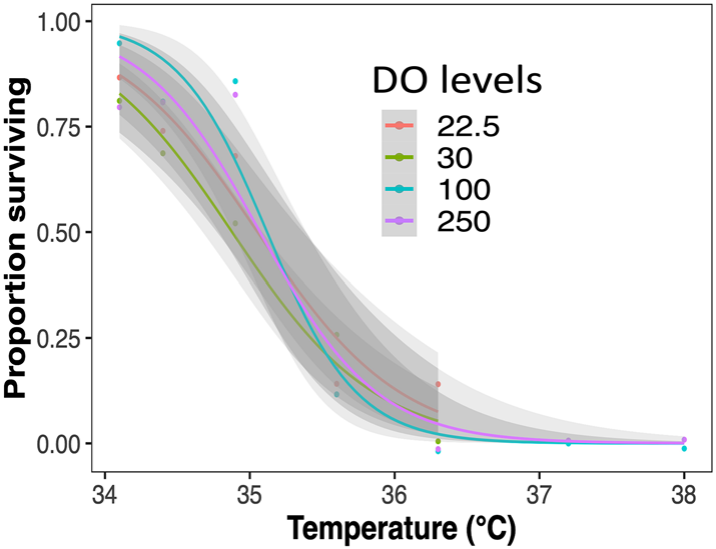
The survival of *Tigriopus californicus* females at different dissolved oxygen levels. Grey shaded areas indicate overlapping 95% confidence intervals.

As expected, there was a significant main effect of temperature on survival of *T. californicus* (χ^2^_1_ = 51.20, *P* < 0.001). Specifically, survival decreased rapidly with increasing temperatures, from an average of approximately 90% at 34.1–34.9 °C to < 10% at 36.3 °C. No females survived at 37.2 and 38.0 °C (Fig. 1). Survival did not differ among low, normal and high DO levels (χ^2^_1_ = 0.72, *P* = 0.40). The interaction of temperature and DO also was not statistically significant (χ^2^_1_ = 0.69, *P* = 0.41). Thus, the temperature-induced mortality of *T. californicus* was independent of oxygen at the levels tested.

### Thermal and oxygen preferences

**Figure 2:**
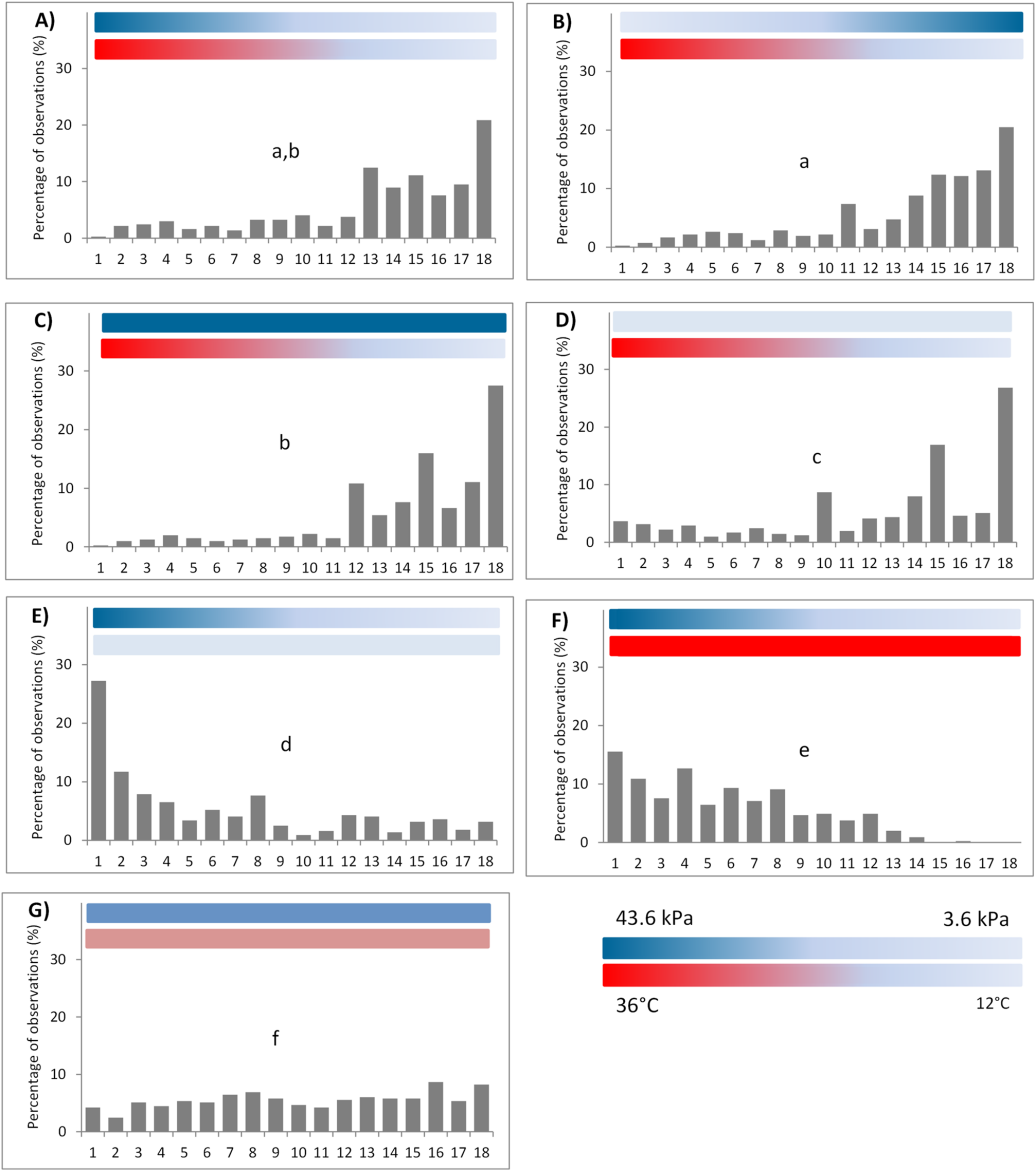
The distribution of *Tigriopus californicus* females in response to gradients of temperature, oxygen, or both within assay chambers. Data are the cumulative percentages (%) of observations in which females were observed in each of the positions from 1 to 18 in the test chambers. The colored horizontal bars within each panel illustrate the ranges of temperature and dissolved oxygen presented in each treatment. Statistical differences (*P* < 0.05) among treatment groups are indicated by different lowercase letters. Panels A–G represent Treatments 1–7, respectively.

Overall, females avoided high temperatures regardless of the oxygen levels in the water (Fig. 2A–D). The distribution of females tended to shift more toward lower temperatures when the oxygen levels were high in this part of the chamber (Fig. 2B,C, pairwise comparisons between treatments 2, 3 and treatment 1, *P* = 0.075 and 0.051, respectively); this pattern was less clear when low DO occurred across the thermal gradient (Fig. 2D, pairwise comparisons of treatment 4 and treatments 1–3, all *P*-values < 0.05). Female distributions were skewed toward high oxygen levels when there was no thermal gradient (Fig. 2E,F, pairwise comparisons between treatments 5 or 6 and 1–4, all *P*-values < 0.001), and this pattern was stronger at 36 °C (Fig. 2F) than at 12 °C (Fig. 2E, pairwise comparison between treatment 5 and treatment 6, *P* < 0.001). When there were no temperature and oxygen gradients, females were distributed evenly in the chambers, and no preferred positions were observed (Fig. 2G, pairwise comparisons between treatment 7 and treatments 1–6, all *P*-values < 0.001). A full list of pairwise *P* values is provided in Table S1 in Supplementary information S2.

### Distance traveled

**Figure 3:**
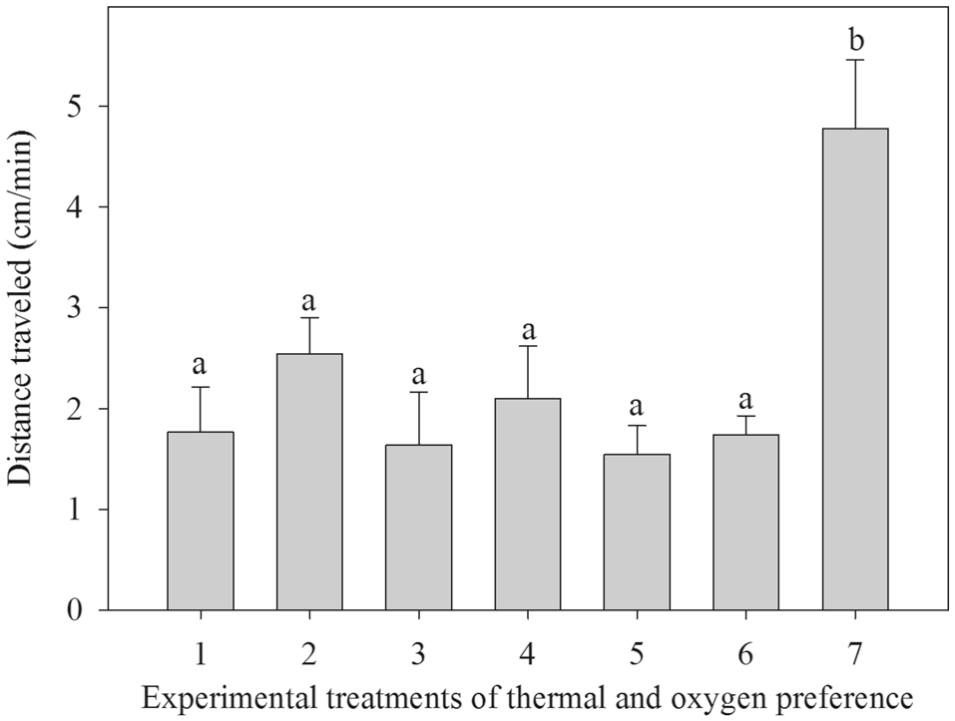
The average distance (cm) traveled per minute by *Tigriopus californicus* females in response to the thermal and oxygen gradients in the test chambers. Data are means + 1 SE. Statistical differences (*P* < 0.05) among treatments are indicated by lowercase letters above the bars.

Copepods were considerably more active in the uniform (no gradients) treatment of intermediate temperature and normal DO conditions relative to all other treatments. For every minute, they traveled 4.8 cm, ca. 2–3 times greater than the average distance traveled by copepods in all other treatments (Kruskal–Wallis, H_6,101_ = 23.30, *P* < 0.001, Fig. 3). There was no difference in distance traveled by copepods among the other treatments (all pairwise *P* values > 0.10, Table S2 in Supplementary information S2).

## Discussion

There is extensive evidence that the splashpool copepods of the genus *Tigriopus* can tolerate extremely high-amplitude fluctuations of environmental conditions^36,37,45,46^. Our results showed strong mortality at temperatures higher than 34.9 °C and only less than 10% of females survived after being exposed to 36.3 °C; no surviving females were observed after exposure to 37.2 and 38.0 °C. These results are comparable to published estimates of the thermal tolerance of *T. californicus* from the same climatic zone^37,38,47^. The thermal tolerance of *T. californicus* populations have been physiologically linked to the ATP synthesis capacity in the mitochondria; ATP synthesis declines at temperatures close to the knockdown temperatures^38^. The level of heat shock protein upregulation also has a positive correlation to thermal tolerance of *T. californicus*^41^. Suppression of a specific heat shock protein (HSPB1) has been shown to reduce the thermal tolerance of *T. californicus* to acute heat stress^17,48^.

Importantly, the overall survival of *T. californicus* did not decrease at two low, but ecologically relevant DO conditions relative to normal DO (~ 100% saturation), regardless of peak exposure temperatures, nor did high DO level mitigate the effects of acute thermal stress. These results appear to contradict predictions of OCLTT^19,21^. A role for OCLTT, particularly during acute heat stress such as imposed in our experiments, is supported by evidence from a range of taxa in both aquatic and terrestrial ecosystems (reviewed in Table 1 in Ref.^21^). However, it is likely that insensitivity of thermal tolerance to low DO levels occurs in species with high capacity to regulate oxygen intake and delivery (e.g., in the snail *Planorbis planorbis*) or when DO levels are not lower than the critical levels (e.g., P_crit_ of 1.1–1.3 kPa for *T. californicus*^50^). For example, the snail *Planorbis planorbis* has 2–4 times higher hemoglobin levels than its congeners^51^; under hypoxic conditions the thermal tolerance of *P. planorbis* was not reduced, but the CTmax of *P. carinatus* was lowered by 1.2–2.1 °C^49^. *Tigriopus* species do not have gills, lack respiratory pigments^50^, and appear to have lost both the transcription factor HIF-1α and oxygen sensing prolyl hydroxylase repressor, EGLN, from their genome^43^. It has been suggested that a high surface-area-to-volume ratio of *Tigriopus* spp. may facilitate oxygen uptake from water^50^. This may allow them to maintain their oxygen consumption rate independent of P_O2_ in the environment until the critical P_O2_ of 1.1–1.3 kPa^50^, which is approximately 4–5 times lower than the lowest P_O2_ of ca. 4.7–5.3 kPa in our experiment. Therefore, the insensitivity of thermal tolerance of *T. californicus* to low, but ecologically relevant DO levels observed in our experiment was in line with previous studies; reduced thermal tolerance of water-breathing species has been observed only at extremely low DO levels^4,26^. As noted above, in the splashpool system, DO levels below P_crit_ of *T. californicus* are exceedingly unlikely to coincide with high temperatures.

**Table 1:** The measured temperatures and dissolved oxygen in the experimental system for the preference behavior test. Data are means ± SD.

Treatments	Header tank 1		Header tank 2		Gradients
	Temperature (°C)	Dissolved oxygen (% saturation)	Temperature (°C)	Dissolved oxygen (% saturation)	
T1	36.7 ± 0.1	198.1 ± 1.9	11.8 ± 0.6	21.0 ± 4.3	Parallel temperature and DO gradients
T2	36.6 ± 0.2	26.4 ± 2.9	12.0 ± 0.3	193.7 ± 1.7	Inverse temperature and DO gradients
T3	36.5 ± 0.2	199.0 ± 2.3	11.7 ± 0.6	198.4 ± 1.9	Temperature gradient. No DO-gradient (high DO)
T4	36.7 ± 0.1	26.9 ± 0.4	11.3 ± 0.5	31.2 ± 2.6	Temperature gradient. No DO-gradient (low DO)
T5	12.2 ± 0.4	198.1 ± 0.4	11.7 ± 0.1	27.9 ± 8.2	No temperature gradient (cool temperature). DO-gradient
T6	36.5 ± 0.1	197.0 ± 9.5	36.3 ± 0.1	31.5 ± 1.2	No temperature gradient (warm temperature). DO-gradient
T7	19.6 ± 0.2	100.1 ± 1.3	19.4 ± 0.3	98.4 ± 2.6	No temperature gradient (intermediate temperature). No DO gradient, normoxic. (control treatment)

Therefore, the oxygen level may not be the limiting factor for thermal tolerance in ecologically relevant scenarios for *Tigriopus* and in similar cases. Instead, the capacity to physiologically cope with other consequences of temperature extremes may be the primary determinant of their thermal tolerance. Mortality under extreme temperatures may be the result of the dysfunction of a number of physiological processes such as the collapse of ATP synthesis^38^, membrane and protein structure instability^52,53^, and suppressed expression of heat shock protein (HSPB1)^17,48^. In light of our whole-organism survival data, we hypothesize that macromolecular disruption, rather than any direct effect of oxygen availability, explains recently published patterns of decreased ATP production capacity in *Tigriopus* mitochondria^38^, particularly because those in vitro experiments appear to have been run under normoxic conditions.

Irrespective of the mechanism(s) for the insensitivity of the thermal tolerance of *T. californicus* to ecologically relevant DO levels, our results join a growing literature suggesting that the OCLTT hypothesis may not be a universal principle for predicting the survivability of species in ecologically relevant conditions in nature^4,26^. Whether the exceptional environmental covariation of temperatures and DO levels in splashpools has selected for high thermal tolerance regardless of DO levels remains to be seen; it is equally plausible that selection for surviving periods of extreme night-time low DO may have coincidentally increased the ability to survive bouts of high temperature using anaerobic ATP production pathways. Recent work reveals that *T. californicus* can survive several days of anoxia^43^. It also is clear that the elevated DO levels that tend to naturally coincide with high temperatures in splashpools^30^ do little to alleviate the effects of high temperature stress on these animals. Although beyond the focus of this study, it will be interesting to explore whether low DO levels may also alter critical thermal minimum (*CT*_*min*_) of *T. californicus* and other species when low DO levels in their habitats occur during cold nights or the winter period^54^. In terrestrial ecosystems, the *CT*_*min*_ values of some insects such as false codling moth *Thaumatotibia leucotreta*^55^, the beetle *Tenebrio molitor*^56^ and crustaceans such as *Porcellio scaber*^56^ are independent of oxygen availability.

Overall, female *T. californicus* demonstrate a strong avoidance of elevated temperatures, with oxygen playing a secondary role in influencing behavior. For example, their high distribution in the low DO region in treatment 1 (Fig. 2A) was likely just to avoid the potentially lethal effects of extreme temperatures in the oxygen-rich water. The distribution of *T. californicus* was considerably more concentrated in oxygen-rich water only when low temperatures coincided with high oxygen (Fig. 2B,C) or there was no thermal gradient in the chambers (Fig. 2E,F). These results suggest that *T. californicus* can avoid low DO conditions.

Interestingly, the genome of *T. californicus* appears to lack prolyl hydroxylase and HIF-1α^43^, but our results clearly illustrate that these animals can sense the relative abundance of oxygen in the water. These results imply the existance of an alternative, extracellular (and perhaps superficial) mechanism(s) for *T. californicus* to sense *p*O_2_. Under uniformly extreme high temperatures, a shift in the distribution of females toward oxygen-rich water was even stronger; none were found in the low DO regions (Fig. 2F). This may be the result of higher basal metabolic demand, indicated by a general higher oxygen consumption rate at higher temperatures in a congener^50^. Finally, *T. californicus* did not show any preferred region within the chambers when they were in the control treatment at room temperature of 19 °C and the DO was maintained at 100% saturation. They also traveled a longer distance in this control treatment than in all other treatments, an indication that our observations of their distributions were consistent throughout the trial and uninfluenced by other confounding factors such as light.

Lastly, the behavioral results complement the physiological results to suggest a potentially novel explanation for how *T. californicus* can thrive in splashpools, where both temperature and DO are highly fluctuating and often extreme. Specifically, the results for thermal tolerance suggest that *T. californicus* may occasionally not be able to physiologically cope with extreme temperature. To survive in splashpools with extremely high temperatures during the day, there must be an alternative mechanism. Interestingly, field observations indicate that splashpools may be highly stratified over their small spatial scales of a few 10 s of centimeters or less (Fig. S1 in Supplementary information S1, Ref.^30^). Indeed, our behavioral preference test showed that females avoided near-lethal temperatures even if by doing so they had to deal with lower oxygen levels at lower temperatures. This behavioral preference of *T. californicus*, which remains to be demonstrated in a natural setting, supports a recent prediction that behavioral responses of natural populations may enable them to exploit microclimatic variations in heterogeneous habitats as an important mechanism to rescue species from rapidly changing environments^17,18^.

Our study provides empirical evidence for the insensitivity of both heat tolerance and thermal preference of the splashpool copepod *T. californicus* to the ambient DO level. These patterns apply in ecologically relevant low and high DO conditions. It is likely that warming may be more stressful for *T. californicus* than low DO levels. Our results are among a small but growing collection of studies showing that the OCLTT hypothesis may not be a universal tool for predicting the thermal tolerance of species, particularly in ecologically relevant scenarios where environmental conditions fluctuate dynamically across a small scale of space and time. The strong behavioral preference of *T. californicus* suggests that together with physiological adjustments^39–41^, microhabitat selection might be used as an alternative mechanism for *T. californicus* to survive in highly fluctuating and often extreme conditions in splashpools^30^.

## Materials and methods

### Study population

Female *Tigriopus californius* were collected in May 2019 from splashpools at Cattle Point Lighthouse (+ 48° 27′ 1.44″ N, − 122° 57′ 48.6″ W) on San Juan Island, WA, USA. The copepods were acclimated to the laboratory condition at 17–18 °C for 2 to 4 months (at least 1–2 laboratory-reared generations). Copepods were fed ad libitum on fish flakes and an irradiated algae mixture (Shellfish Diet 1800, Reed Mariculture), each provided once a week. They were kept under a photoperiod of 13L:11D (light:dark cycle). Salinity values correspond to the practical scale of 32.5–42.0 and dissolved oxygen (DO) was maintained above 80% of the saturation level (> 6 mg L^-−1^) throughout the acclimation period. Salinity and DO were measured using a YSI digital meter (Pro 2030, Yellow Springs Instruments, USA).

### Thermal tolerance assay at different dissolved oxygen levels

The thermal tolerance of *T. californicus* was quantified based on survival after exposing females to peak temperatures of 34.1, 34.4, 34.9, 35.6, 36.3, 37.2 and 38.0 °C at different DO levels. Specifically, females carrying egg masses (380 individuals, n = 15–16 per temperature × DO combination) were randomly collected from the culture and exposed to a 5 h heat ramp at one of the peak temperatures (34.1–38.0 °C) at each of four DO levels: 22.5, 30, 100 and 250% of the oxygen saturation level. Both DO and peak temperatures are ecologically relevant to the splashpools at the collection site. Individual females were placed in 0.2 mL PCR tubes (conical shape, h = 20.8 mm and d_top_ = 5.46, d_bottom_ = 2.8 mm) filled with 150 µL of the appropriate seawater (32 ppt) and DO level. Copepods could swim freely inside the tubes during the test, and they exhibited typical swimming behavior after the test.

To create different DO levels in the PCR tubes during the heat ramp, we prefilled tubes with seawater adjusted to one of the desired levels. Dissolved oxygen levels were manipulated in a 20 L water bath using a custom-built, Arduino microcontroller system that regulated DO (while maintaining a constant pH of ~ 8.05) by coordinating the opening/closing of solenoid valves connected to oxygen, carbon dioxide, and nitrogen gas cylinders. The systems included calibrated temperature, DO (Honeywell DL5000), and pH (Honeywell Durafet III) sensors connected to a Honeywell UDA1282 Universal Dual Analyzer. The milliamp outputs of this analyzer provided feedback to the Arduino on the current conditions in a header tank; after comparing the current conditions to the desired setpoints, the Arduino triggered brief (10 s of milliseconds) pulsed openings of the solenoid valves to regulate gas flow. This cycle continued on a continuous loop, constantly monitoring and maintaining the DO level. Target DO levels (in mm Hg) were confirmed in the PCR tubes using a fiberoptic oxygen sensor (Neofox, Ocean Optics) prior to the tubes being capped and sealed with parafilm. To sustain these DO levels during the heat ramp, the entire thermocycler used for the thermal tolerance assay was housed in a sealed incubator, in which we manipulated the atmospheric oxygen levels in parallel with the desired DO in the seawater by pumping nitrogen or oxygen into the incubator. This arrangement was necessary because in preliminary trials all DO levels equilibrated with the atmosphere by the mid-way point of the 5-h heat ramp. Due to safety concerns around high-amperage electrical equipment, we could only increase oxygen levels in the incubator to 150% saturation. Using the incubator, DO levels within the PCR tubes were 4.7–5.3, 5.3–6.4, 21.2–21.3, and 50.7–53.3 kPa (22, 30, 100 and 250% saturation), respectively, at the start of the thermal tolerance assay. We confirmed in preliminary trials that these target DO levels in PCR tubes (each containing one female copepod) were maintained during the heating phase until reaching the peak temperature, but they drifted by the end of the assay. At the completion of the heat ramp, DO levels in the PCR tubes were 12.7–13.3, 14.0–14.4, 21.2–21.3, and 40–42.7 kPa (60–62, 66–67.5, 100, 188–200% saturation, respectively). Thus, although DO conditions did not remain constant for the entire duration of the heat ramp, they remained different from each other. Both low DO treatments (22.5 and 30% saturation) remained within the ecologically relevant low DO ranges found in splashpools during the heating phase^30^. Experimental assays were run using identical methods as these preliminary trials, but we did not measure DO levels in PCR tubes for experimental copepods.

For each heat ramp, PCR tubes were placed in an Eppendorf Mastercycer gradient thermocycler, which was custom-programmed to generate a gradual rise and fall of temperature over a five-hour period. The start temperature for the heat ramp was 20 °C. The thermocyler was programmed to a new setpoint every 10 min. Over the first 25 min all columns increased by 1 °C. Subsequent setpoints were programmed to increase 1 °C every 10 min to a preset peak temperature of 37 ± 3 °C at 185 min. Using the gradient feature, each column reached a unique peak temperature of 34.1, 34.4, 34.9, 35.6, 36.3, 37.2 or 38.0 °C. Upon reaching the peak, the temperature was maintained for 1 h. Following the 1-h exposure, temperature was decreased to 20 °C over the course of one hour. This protocol created a thermal profile more similar to the environmental temperature variation experienced by *T. californicus* in the wild^39^. Preliminary trials revealed that females exposed to low DO conditions and a peak temperature of 36 °C suffered 100% mortality. In order to reduce unnecessary use of animals, it was determined that low DO levels, 22.5 and 30% saturation, combined with peak temperatures of 37.2 and 38.0 °C would not be tested as part of this experiment. Therefore, a total of 40 females were tested at each of the low DO levels of 22% and 30%, and 56 females were tested at the normal and high levels. This heat-ramp procedure was repeated twice at each dissolved oxygen level. DO levels were randomized and only one was examined per day. The survival of females was checked immediately after the ramp and daily for the following 4 consecutive days. Mortality was determined when females were unresponsive to mild shaking of the vial, changed colors to bright red, and the urosome was bent sharply at a right angle to the cephalothorax^57^. Statistical analyses were conducted using survival at day 4.

### Behavioral preference assay in the presence of temperature and oxygen gradients

**Figure 4:**
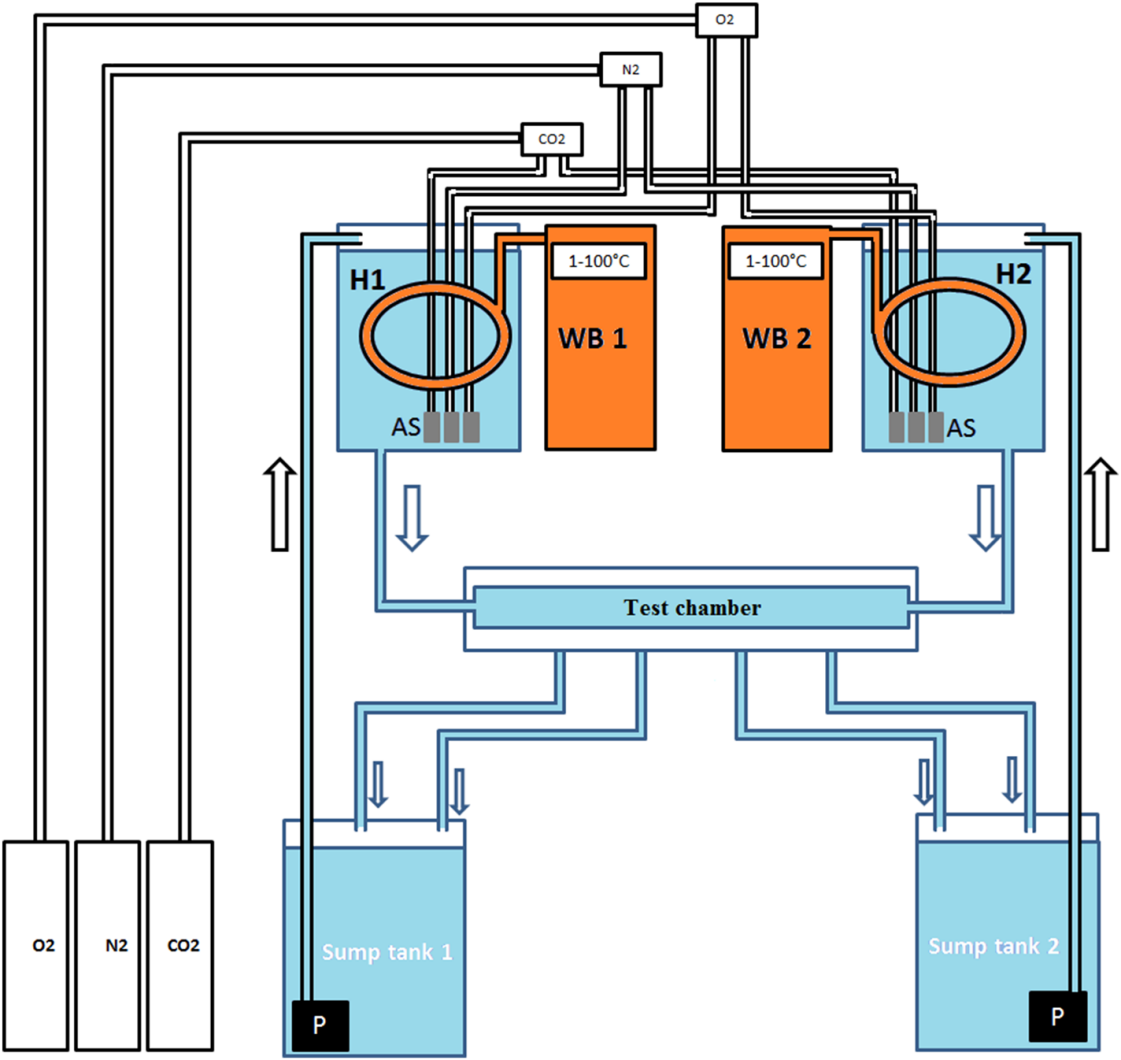
Experimental system for the behavioral preference assay, in the presence of oxygen and/or temperature gradients. H1 and H2 are header tanks where both temperatures and dissolved oxygen can be independently controlled within the ranges of 1–100 °C and 0–200% saturation, respectively. WB1 and WB2 are water baths where temperatures were set up to control temperatures in the H1 and H2 tanks, respectively. Gases (O_2_, N_2_, and CO_2_) were fed from cylinders to each header tank via airstones, and an Arduino-controlled system pulsed gas flows independently to each header tank through solenoid valves to maintain pH and *p*O_2_ at desired levels. AS = Airstones and P = pump. Arrows indicate the directions of water flow.

To test whether DO may impact thermal preference of *T. californicus*, we determined the positions and distance traveled by egg-mass bearing females (n = 14–15 individuals per treatment) in each of 7 treatment conditions (Table 1) in preference chambers (Fig. 4). Preference chambers were in-house designed based on the system for *Daphnia magna* described in Zeis et al.^58^, with extensive modifications (Fig. 4). Specifically, 5 identical chambers were constructed of acrylic (L × W × H = 22.86 × 1.27 × 1.27 cm, volume = 36.87 ml). The chamber was sealed with a rubber gasket and acrylic lid. It was divided into 18 equal intervals, 1.27 cm each, by marking the outside of the chamber. Copepods could swim freely throughout the chamber without any physical barriers. There were inlets at each end, which connect to two different header tanks (size: L × W × H = 28.58 × 31.12 × 40 cm; volume = 20.8 L) where the water temperature and DO were controlled by two programmable, recirculating water baths and the N_2_ and O_2_ gas systems. The pH in the water was controlled at around 8.05 ± 0.05 by the CO_2_ gas system. Gas flows were automatically controlled by an in-house Arduino microcontroller system as described for the thermal tolerance assays above. Each preference chamber has 4 outlet ports distributed equally along one side (the distance between two adjacent outlet ports is 4.57 cm); inlet and outlet ports were fitted with a 50 µm mesh screen to prevent copepods swimming out of the chamber. The outflow rates were 0.8–0.9 mL/min for outlets. This resulted in a total of roughly 3.25 mL water outflow per min, approximately 9% of the volume of the preference chamber. This flow rate is comparable to an assay for *Daphnia magna*^58^. Copepods swam freely throughout the preference chambers, therefore the flow did not impede the swimming or behavioral preferences of *T. californicus*. Two outlet ports from each side of the chamber were connected to one sump tank and two others were connected to a second sump tank. The sump tanks have the same size and volume as the two header tanks. The water in each of the sump tanks was pumped up to the corresponding header tank to create two closed and recirculating water systems, which could be regulated independently for temperature and DO.

Prior to an experiment, the desired temperature (11 to 37 °C) and DO levels (0–200% saturation) were established in the two header tanks using recirculating water baths and the Arduino system, respectively (see Table 1). In the chambers, we confirmed the temperature and DO gradients at 6 positions; temperature fluctuated 34.5–35.1 °C at the high end; 28.3–30.6, 25.5–27.1, 18.5–22.7, 15.7–18.4 °C at the four outlet ports; and 13–13.4 °C at the low end. Similarly, observed DO ranges from the high to low DO ends of the apparatus were 51.7–62.5, 26.4–53.3, 21.9–26.4, 9.2–14.7, 4.8–8.0 and 0.7–3.2 kPa, respectively. These are also ecologically relevant for the temperature and DO levels observed in splashpools occupied by *Tigriopus* (Fig. S1—Supplementary information S1, Ref.^30^). For each experimental run, we randomly collected five egg-mass carrying females from the lab culture (temperature of 17–18 °C) and assigned one individual per preference chamber. In preliminary trials, we observed that copepods tended to modify their behavior in the presence of a con-specific, so our protocol isolated the effects of environment from any social factors.

Females were allowed 10 min to explore the chambers before beginning data collection. Our preliminary observations showed that females swam freely within the first three to five minutes, and subsequently showed a more stable position in the chamber. The female positions in each chamber were observed once per min for 29 min (30 observations). All preference assays were conducted in diffuse light to avoid stressing the animals, and the orientation of the gradients relative to the room was randomly reversed for some chambers to avoid systematic influence of the surroundings on copepod behavior.

The distance traveled by a female during the observation period was calculated by summing the distances from one observation to the next. This cumulative measure of distance traveled may not be accurate for two reasons. First, it captures movements in increments of 1.27 cm; second, some females may move forward and back several times between consecutive observations. Nonetheless, this method provides a rough estimate of how active each female was within the observation chambers. The distance traveled was standardized to units of cm per minute, as not all females had all 30 observations due to the difficulty of observing them in the chambers under dim light. Specifically, 96/101 (95%) behavioral assays had 27–30 observations. The other five behavioral assays (5%), each had 10, 18, 21, 24 and 26 observations.

### Statistical analyses

To test for the effects of peak extreme temperatures, DO levels and their interaction on the thermal tolerance of *T. californicus* females, we ran a generalized linear model in R, using a binomial link function (0 = dead, 1 = alive). Temperature and DO were included as fixed factors. For thermal and oxygen preferences, the number of times that a female was observed in a specific position in the gradient (1–18 in the chamber) was counted to calculate the percentage of time in each position. The distribution of *T. californicus* in each position in the preference chambers is the main indicator of thermal and oxygen preferences. A Chi-square test was employed to test for pairwise differences in the distribution of females among treatments. For the distance traveled by females per minute, data were initially checked for normality using a Shapiro–Wilk test and the homogeneity of variance using Levene’s test; both assumptions for ANOVA were not met (*P* < 0.05). Therefore, we used a non-parametric Kruskal–Wallis test to examine differences in the distance that copepods traveled per minute. *P* values < 0.05 are considered statistically significant. All analyses were run in *R* (v.3.1.3).

### Data deposition

Data for this study are available via the WSU Research Exchange 10.7273/gjg0-p174.

## Supplementary information

Supplementary Information.
