# Supplementary material for: Heat tolerance and thermal preference of the copepod Tigriopus californicus are insensitive to ecologically relevant dissolved oxygen levels
Source: Sci Rep. 2020 Nov 3;10:18885. doi: 10.1038/s41598-020-75635-z (PMC7641137; doi:10.1038/s41598-020-75635-z)
Supplement: Supplementary file 1 — Supplementary Information. [file 41598_2020_75635_MOESM1_ESM.docx]

**Heat tolerance and thermal preference of the copepod *Tigriopus californicus* are insensitive to ecologically relevant dissolved oxygen levels**

Khuong V. Dinh^1,*^, Arani Y. Cuevas-Sanchez^1^, Katherine S. Buhl^1^, Elizabeth A. Moeser^2^, W. Wesley Dowd^1^

^1^Washington State University, School of Biological Sciences, P.O. Box 644236, Pullman, WA 99164-4236, USA

^2^University of Southern California, Department of Environmental Studies

Corresponding author: Khuong V. Dinh, email: [khuong.dinh@wsu.edu](mailto:khuong.dinh@wsu.edu)

Telephone number: +1 (509) 339-4903

**Supplementary information S1: Thermal stratification in the splashpool**

We present an example of vertical thermal stratification within a splash pool in Figure S1. In July and August 2015 we deployed a set of iButton temperature data loggers (Maxim DS1921, measurement resolution of 0.5°C, recording interval 20 min) vertically within a small splashpool at Hopkins Marine Station, Pacific Grove, California, USA. The loggers were encased in wax and affixed to an acrylic stick with elastic bands. The loggers were spaced evenly from the bottom to the surface of the pool. There were 7 loggers at the start of the experiment, but the top 2 were exposed by the end of the experiment due to evaporation. As a result, this plot illustrates the difference between depth 3 (third from the top) and depth 7 (bottom of splashpool) over a period of 18 days. A positive number indicates that the near-surface water was warmer than the bottom; these events coincide with midday in the time-series.


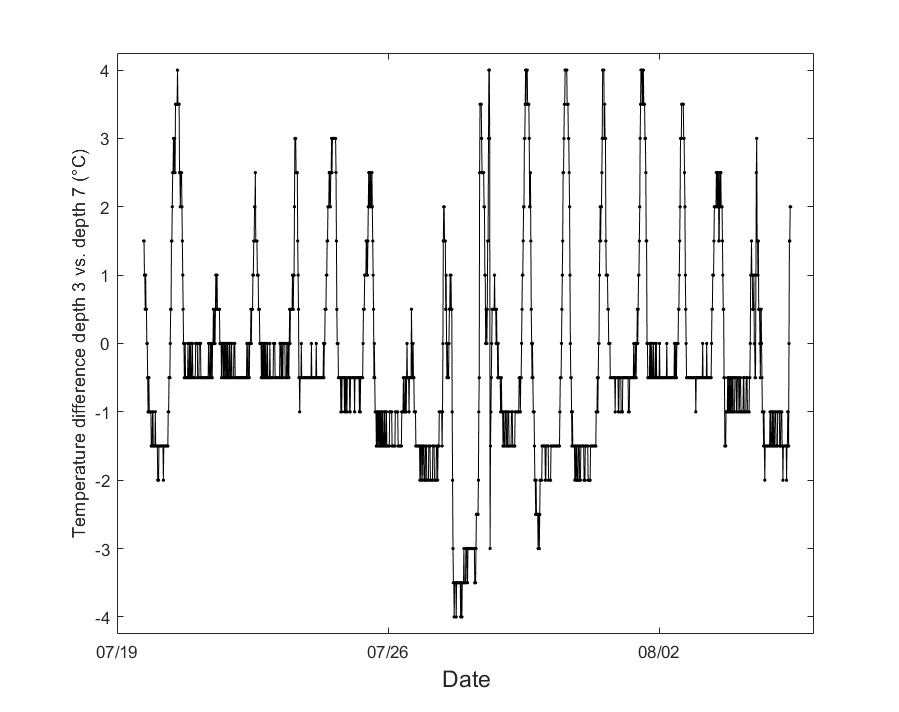


Figure S1. Thermal stratification in the splashpool (36.62178° N, 121.90438° W).

**Supplementary information S2**: Statistical tables

**Table S1**. Statistical pair-wise *P* values of a Chi-squared test for the effects of thermal and dissolved oxygen gradients on the behavioral preference of *Tigriopus californicus*. Details of treatments T1-T7 are in Table 1.

| Treatments | T1 | T2 | T3 | T4 | T5 | T6 | T7 |
| --- | --- | --- | --- | --- | --- | --- | --- |
| T1 |  | 0.075 | 0.051 | 3.2×10^-3^ | 1.1×10^-48^ | <0.001 | 6.9×10^-6^ |
| T2 |  |  | 8.1×10^-5^ | 1.3×10^-6^ | 8.1×10^-60^ | <0.001 | 1.2×10^-7^ |
| T3 |  |  |  | 2.0×10^-2^ | 3.8×10^-72^ | <0.001 | 1.1×10^-14^ |
| T4 |  |  |  |  | 9.5×10^-3^ | <0.001 | 4.8×10^-12^ |
| T5 |  |  |  |  |  | <0.001 | 2.4×10^-30^ |
| T6 |  |  |  |  |  |  | 1.3×10^-16^ |
| T7 |  |  |  |  |  |  |  |

**Table S2**. Statistical results testing for the effects of thermal and dissolved oxygen gradients on the average distance traveled by *Tigriopus californicus* per minute in each of seven treatments. Values are from multiple comparisons of Kruskal Wallis test. Statistically significant pair-wise differences are noted with asterisks (* P < 0.05, ** P < 0.01, *** P < 0.001).

|  | T1 | T2 | T3 | T4 | T5 | T6 | T7 |
| --- | --- | --- | --- | --- | --- | --- | --- |
| T1 |  | 1.000000 | 1.000000 | 1.000000 | 1.000000 | 1.000000 | 0.029880* |
| T2 |  |  | 0.704913 | 1.000000 | 1.000000 | 1.000000 | 0.005968*** |
| T3 |  |  |  | 1.000000 | 1.000000 | 1.000000 | 0.000837*** |
| T4 |  |  |  |  | 1.000000 | 1.000000 | 0.039559* |
| T5 |  |  |  |  |  | 1.000000 | 0.003606** |
| T6 |  |  |  |  |  |  | 0.026778* |
| T7 |  |  |  |  |  |  |  |
